# Microbiota and the gut-brain-axis: Implications for new therapeutic design in the CNS

**DOI:** 10.1016/j.ebiom.2022.103908

**Published:** 2022-03-04

**Authors:** Longsha Liu, Jun R. Huh, Khalid Shah

**Affiliations:** a Center for Stem Cell and Translational Immunotherapy (CSTI), Brigham and Women's Hospital, Harvard Medical School, Boston, MA 02115, USA; b Department of Neurosurgery, Brigham and Women's Hospital, Harvard Medical School, Boston, MA 02115, USA; c Department of Immunology, Blavatnik Institute, Harvard Medical School, Boston, MA, USA; d Evergrande Center for Immunologic Diseases, Harvard Medical School and Brigham and Women's Hospital, Boston, MA, USA; e Harvard Stem Cell Institute, Harvard University, Cambridge, MA 02138, USA

**Keywords:** Gut microbiome, Gut-brain-axis, CNS diseases, Glioblastoma, Therapy

## Abstract

The recent revelation that the gut microbiome, home to approximately 100 trillion microorganisms, is implicated in the development of both health and disease has spurred an exponential increase in interdisciplinary research involving gut microbiology. In all this hype, there is a need to better understand and contextualize the emerging evidence for the role of the gut microbiota in neurodegenerative and neurodevelopmental diseases, including central nervous system (CNS) malignancies. In this review, we aim to unravel the complex interactions of the microbiota-gut-brain-axis to pave a better understanding of microbiota-mediated pathogenesis, avenues for noninvasive prognosis, and therapeutic possibilities leveraging microbiota-gut-brain-axis modulations. We further provide insights of the ongoing transition from bench to bedside and discuss limitations of current approaches. Ultimately, we urge the continued development of synergistic therapeutic models with considerable consideration of the many gut-resident bacteria that will enable significant progress for the treatment of many neurological diseases.

## Introduction

The gut microbiome comprises a vast ecology of commensal bacteria, archaea, fungi, and viruses in and on the body.^1^ However, understanding both the nature and function of the microbiota is still very much in its infancy as the exact composition varies across each individual and there is no definitive understanding on what truly constitutes a healthy adult gut microbial profile beyond exhibiting both diversity and stability. Due to this wide variety in microbiota composition, functional capacity, defined by metabolic pathways, may be a better metric for the health of one's microbiome provided the conservation of basic metabolic pathway categories across individuals.^1^ Through studies observing deficits and dysfunctionalities in germ-free (GF) mice, it has now become apparent that the microbiota modulates various vital physiological activities including synthesizing metabolites, fermentation of indigestible carbohydrates, and, importantly, regulation of the immune and nervous systems.^2^ Overall, there is compelling evidence that the microbiota is fundamental to physiological homeostasis and, consequently, in shaping and maintaining the immune system. Moreover, the gut microbiota holds significant potential as a reservoir for new therapeutic opportunities, with the exciting possibility of targeting and impacting the microbiota-gut-brain axis for treating many challenging diseases in the central nervous system.

## The microbiota-gut-brain axis

**Figure 1:**
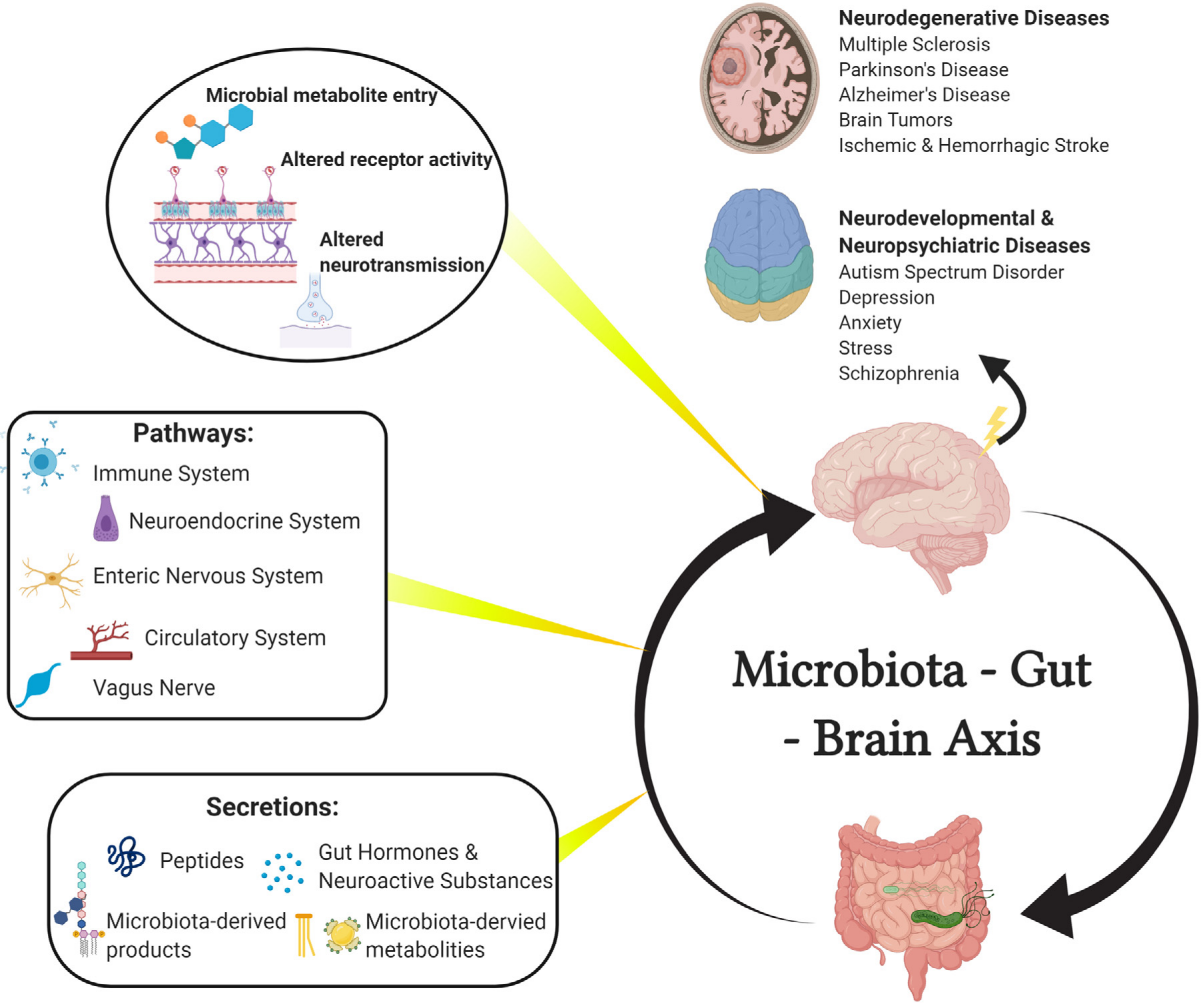
The microbiota-gut-brain axis. The bidirectional communication between the brain and gut microbiota is mediated by several pathways including the immune system, neuroendocrine system, enteric nervous system (ENS), circulatory system, and vagus nerve. The routes of these pathways contain various neuroactive compounds including microbial-derived metabolites, microbial-derived products, peptides, gut hormones, and neuroactive substances. Upon entering the brain, metabolites can subsequently influence neurodevelopment and neurodegeneration of numerous conditions, such as multiple sclerosis, Parkinson's Disease, Alzheimer's Disease, CNS malignancies, stroke, autism spectrum disorder, depression, anxiety, stress, and schizophrenia.

The signaling mechanism behind the microbiota-gut-brain axis communication is of particular interest when considering therapeutic design. The brain modulates gut function through the hypothalamic-pituitary-adrenal axis and the autonomic nervous system; norepinephrine, for example, is released by the brain during stress and has been found to stimulate gut pathogen proliferation.^3^ Conversely, the gut modulates CNS functions through involving a variety of microbiota-derived metabolites and products, neuroactive substances, and gut hormones that traverse through the enteric nervous system, vagus nerve, circulatory system, or immune system to reach the brain (Figure 1).^4^ Collectively, these pathways are referred to as the microbiota-gut-brain axis (MGBA).

Notably, of the many pathways possible, the vagus nerve is the most direct route as different receptors on the vagal afferents detect then transmit stimuli from the gut to the brain. In fact, the vagus nerve was recently implicated in CNS mood and behavior via influencing CNS reward neurons.^5^ Moreover, the gut-immune-brain pathway and its importance to overall physiological function cannot be understated as the microbiome is intimately involved in the induction, training, regulation, and function of not only local immunity but also systemic innate and adaptive immune response. This has been supported in pre-clinical models, as GF mice exhibit significant deficiencies in immunity including increased susceptibility to infections via reduced size and functionality of Peyer's patches, absent mucous layer, altered IgA secretions, and reduced secretion of proinflammatory Th cells.^6^^,^^7^

**Microbial-derived metabolites and products** are major contributors in the MGBA that exert their effects primarily through receptor-mediated interactions on various host tissues or cells. One of the most well-characterized metabolites are short-chain fatty acids (SCFAs) and endogenous tryptophan. SCFAs, a derivative of microbial-induced decomposition of carbohydrates, have been suggested to contribute to glucose homeostasis, mucosal serotonin release, lymphocyte function, and influence learning and memory acquisition via preservation of brain integrity.^7^ Although studies are scarce, it is suggested that SCFAs may even be able to bypass the blood brain barrier (BBB) as the metabolite is detectable in human cerebrospinal fluid and SCFA uptake was experimentally observed in rats following injection of ^14^C-SCFAs into the carotid artery.^8^ To further support the role of SCFAs in CNS homeostasis, GF mice were noted to have increased BBB permeability; however, recolonization of the same mice with SCFA-producing bacteria recovers BBB integrity.^9^ Mechanistically, studies have reported interactions between SCFAs with G-protein coupled receptors (GPR) in enteric nerves (GPR41) or adipose tissues (GPR43), among others, for a variety of different functions.^10^ It is worthy to note, however, that findings have been inconclusive; for example, acetate, a key SCFA produced by gut bacteria, has been suggested to regulate food consumption in one study^11^ but implied to stimulate food intake via ghrelin release in another.^12^

Microbial-induced metabolism of dietary tryptophan to indole compounds also bears mention as some bacteria, particularly certain species of the Lactobacillus genus, were recently identified to play key roles in activating the aryl hydrocarbon receptor and, consequently, in cell cycle regulation and T-cell differentiation.^13^ Importantly, studies have demonstrated the essential role that dietary tryptophan plays in encephalitogenic T cell responses, thereby inducing CNS autoimmunity.^14^ However, studies investigating into the importance of tryptophan via tryptophan depletion are still presently contentious in findings, with one, for instance, reporting impaired cognition^15^ while another reported enhanced cognition.^16^

**Microbial-derived products** also play major roles in the MGBA communication, often through interactions with the toll-like receptors in the ENS and CNS that can sense the products through recognized molecular patterns. Lipopolysaccharide (LPS), a component released by gram-negative bacteria, for example, has been shown to be recognized by toll-like receptors 4, which are primarily expressed on microglia of the CNS, that subsequently induces proinflammatory cytokine production and proliferation.^17^ Notably, this induced immune response has led to neuroinflammation, microglial activation, and neuronal cell loss that resulted in cognitive impairments and correlated with anxiety and depression.^18^ Polysaccharide A, another common microbial-derived product, is secreted by *B. fragilis* and is recognized by toll-like receptors 2 that then induce a protective CNS anti-inflammation response.^19^

Overall, the interactions of microbial-derived metabolites and products within the MGBA is abundantly diverse and complex where one metabolite may interact with multiple receptors spanning multiple tissue and cell types, resulting in an array of different physiological, immune, and CNS responses. Looking forward, therapeutic intervention with SCFAs may be a particularly promising candidate and studies, though still somewhat inconsistent, are already underway in models of autism spectrum disorder,^20^ Alzheimer's disease,^21^ Parkinson's disease,^22^ and multiple sclerosis.^23^

**Gut hormones** are also important to consider in gut-brain signaling. Casual links have been observed between obesity and mood disorders, further reinforced by correlations of several gut hormones (i.e., CCK, ghrelin, and 5-HT) with anxiety and depression.^24^ 5-HT is one of the most well-characterized examples of gut hormones with a wide diversity in receptor subtype and location; when produced by the enterochromaffin cells (EECs), 5-HT has been observed to promote cytokine secretion from lymphocytes and monocytes as well as send signals to the CNS via activating vagal sensory afferents.^25^^,^^26^ Consequently, it bears mention that the microbiota is heavily implicated in the production and release of gut hormones; GF mice, for example, have significantly reduced levels of 5-HT and dopamine compared to normal and GLP-1 secretion was found to be promoted through indirect interactions involving LPS and SCFAs, respectively.^27^^,^^28^ It is important to note, however, that gut hormones may also affect the microbiota; 5-HT, for instance, can be released by EECs and secreted towards the gut lumen, thereby altering the gut microbial profile.^29^ Therefore, moving forward, it is important to separate the cause and effect of the gut hormone-gut microbiota relationship and contextualize the findings to guide CNS therapeutic design.

**Neuroactive molecules** represent another class of microbiota-affiliated molecules that also influence the MGBA through likely the ENS. Gut microbes have been recognized to modulate, if not directly synthesize, neuroactive substances such as dopamine, noradrenaline, acetylcholine, histamine, melatonin, and gamma-aminobutyric acid that, in turn, modulate the CNS.^30^ It is still not well known, however, how these neuroactive molecules affect the CNS as they are unable to bypass the BBB. Further investigation into the pathways of gut-modulated neuroactive substances is needed.

**Figure 2:**
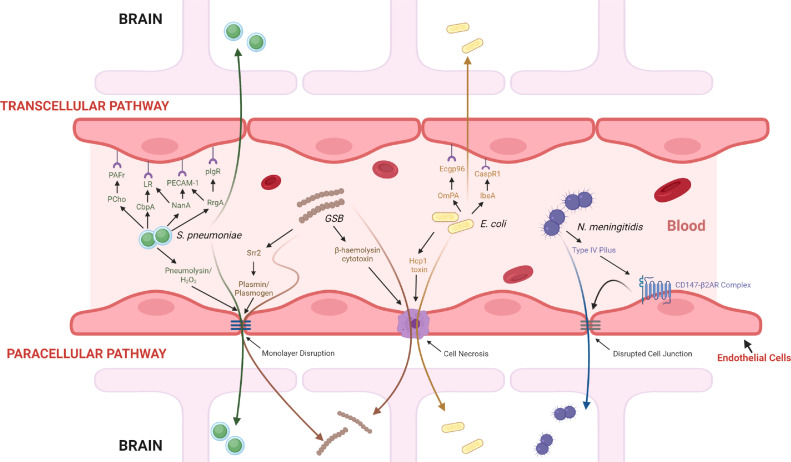
Mechanisms for bacteria crossing the blood brain barrier. The mechanisms for four common meningitis-causing bacteria have been elucidated in recent years through a mixture of largely *in vitro* and *in vivo* data. While a pre-requisite for these bacteria is the survival in the bloodstream long enough to interact with brain endothelial cells, the mechanisms and pathways differ. *N. meningitidis* is known to use a paracellular pathway that involves the bacteria adhering the endothelial cells through the binding of type IV pili to the CD147-β2-adrenergic receptor (β2AR) complex.^33^ This interaction activates the complex and induces membrane protrusions to form that allows increased bacterial resistance to the forces of blood flow and subsequently enables vascular colonization. Moreover, the activation induces signaling events that lead to a disrupted cellular junction, allowing bacteria to cross paracellularly. *E. coli* K1 has been documented to possess mechanisms that enable them to enter the BBB both paracellularly and transcellularly. *E. coli* bacteria can cross paracellularly through a cell necrosis event that is induced by the bacterial secretion of haemolysin co-regulated protein 1 (Hcp1), which causes apoptosis in endothelial cells.^35^ Alternatively, bacterial transcytosis across the endothelium can occur through the adhesin bindings of OmPA (Outer membrane protein A) and IbeA to the receptors Ecgp96 and CaspR1, respectively.^34^ Group B *Streptococcus* (GBS) are predominately documented to possess paracellular mechanisms for entry. The bacterial serin rich repeat protein 2 (Srr2), for example, can promote cell wall degradation through interactions with plasminogen and plasmin, and ultimately result in endothelial monolayer disruption.^36^ Alternatively, the production of β-haemolysin is cytolytic to brain endothelial cells and can form pores in the BBB that ultimately also lead to cell necrosis. Lastly, *Streptococcus pneumoniae* can either enter paracellularly through endothelial monolayer disruptions caused by the bacterial production of pneumolysin and H_2_O_2_ or transcellularly via several interactions: PCho (bacterial phosphorylcholine) with PAFr (platelet-activating factor receptor); CbpA (choline-binding protein A) to LR (laminin receptor); NanA (neuraminidase A) to LR and PECAM-1 (platelet endothelial cell adhesion molecule-1); and RrgA to PECAM-1 and plgR (poly immunoglobulin receptor).^37^, ^38^, ^39^

**Direct microbial invasion beyond the BBB** is also possible, although the specific mechanisms and exact point of entry remains poorly understood. In general, while the duration and minimum level of bacteria required to cause bacterial invasion varies among the different pathogens, a common prerequisite for bacterial pathogenesis is often the asymptomatic colonization of respiratory, pharyngeal, or digestive mucosal surfaces followed by a sustained survival in the bloodstream – resisting or escaping phagocytosis and other immune responses – before interacting with brain endothelial cells and finally breaching the BBB through various pathways.^31^ Following bacterial adhesion to brain endothelium, bacteria could traverse the endothelium through a transcellular pathway via brain endothelial cells, a paracellular pathway via disrupting intercellular junctions, or even a Trojan-Horse mechanism via infected phagocytes.^32^ Some pathogenic bacteria and their plausible pathway(s) are summarized in Figure 2.^33^^–^^39^ It is important to consider, however, that most of the findings remain highly speculative in nature. For one, the relevance and translatability of *in vitro* data to *in vivo* adhesive characteristics of pathogens is still questionable since bacteria must withstand blood flow *in vivo*. Nonetheless, bypassing the BBB remains a prevalent clinical challenge for delivering drugs to treat neurological diseases, so understanding how some bacteria succeed via direct interactions with the CNS barriers is both clinically relevant and important when designing therapies.

## Microbiota-gut-brain axis dysregulation: CNS disorders

There has been increasing evidence implicating the imbalance of the microflora in the development of various neurodevelopmental and neurodegenerative diseases. As several reviews have already comprehensively addressed this, the associations are only briefly discussed.^40^^,^^41^

Parkinson's disease (PD) is a well-studied example where the initiation of PD etiology is suspected to begin in the gut, with emerging evidence of α-synucleinopathy originating in the ENS during early stages of PD progression.^42^ Interestingly, nearly 80% of individuals with PD experience constipation issues that often precede PD diagnoses by several years.^43^ Furthermore, some gut bacteria have even been validated as highly sensitive biomarkers for PD diagnosis, such as *Prevotellaceae*, which was found to sharply decline following PD onset, and *Enterobacteriaceae,* whose increased abundance was found to positively correlate with PD symptom severity.^44^ Remarkably, a recent model for diagnosing PD utilizing *Prevotellaceae* abundance and constipation status boasted a specificity of 90.3%.^44^

Alzheimer's disease (AD) etiology has also been strongly linked to the microbiota; metabolic molecules from microbiota have been associated with phosphorylated tau/Aβ42 AD biomarkers, amyloid-β load, and activation of the NLRP3 inflammasome pathway.^45^^,^^46^ The buildup of Aβ deposition in turn catalyzes release of various proinflammatory molecules that causes the neuroinflammation in AD pathology.^46^ *H. pylori*, for example, has been reported to induce hyperphosphorylation of tau protein as well as secretion of inflammatory mediators and amyloids^47^; subsequently, treatment of *H. pylori* infection via triple eradication therapy was observed to cause improved cognitive parameters in AD patients.^48^

Multiple sclerosis (MS) and autism spectrum disorder (ASD), though not as extensively elucidated with regards to associations with the gut microbiome, also bear mention. A clinical trial investigation of gut microbial profile in MS patients, for example, has revealed elevated levels of specific microbial taxa including *Akkermansia muciniphila* and *Acinetobacter calcoaceticus* that were later observed *in vitro* to contribute to increased proinflammatory T-cell response and weakened Tregs activity.^49^ Such findings have been reinforced in other correlative studies, including linking increased levels of *Fusobacteria* with future MS relapse possibilities.^50^ Furthermore, mice that received fecal microbiota transplantation (FMT) from MS patients showed worse experimental autoimmune encephalomyelitis (EAE) phenotypes and fewer anti-inflammatory regulatory T-cells than mice that received FMT from healthy individuals.^49^

Meanwhile, studies have also noted considerable microbial composition differences between individuals with ASD and neurotypical individuals, including a decreased abundance of Bifidobacterium, considered a beneficial genus, and an increased abundance of Clostridia, a potentially pathogenic genus.^51^ Moreover, FMT from healthy individuals into ASD patients resulted in moderate improvements to gut comorbidities (e.g., constipation and abdominal pain) and behavior (e.g., repetitive behavior and social skills deficits).^52^

Lastly, brain tumors are also speculated to be impacted in their initiation or progression by the microbiome via an altered immune system, microbial-induced genotoxic effects, or signaling/proliferation pathways changes.^53^ A recent study comparing fecal samples from glioma patients and mouse models with healthy controls revealed significant differences in beta diversity, the ratio of *Firmicutes* to *Bacteroides*, and relative abundance of *Verrcomicrobia* and *Akkermansia.*^54^ Conversely, the same study demonstrated that such microbial differences became significantly, if not fully, diminished in mice upon treatment with temozolomide, demonstrating its translational capabilities. Moreover, it has been found that appropriate microbiota activity reduces immune suppression and improves responses to immunotherapies for glioma models.^55^

More broadly, while the implication of the microbiota in CNS diseases is of significant interest for reshaping therapeutic design, gut-modulated physiological and cellular changes are likely extremely complex and multifaceted. Future studies will need to investigate the translatability of correlative observations and verify the reproducibility of existing experimental findings. It is also important to consider that pre-clinical and clinical experiments for CNS diseases often involve confounding variables such as antibiotics and medication that, when coupled with other factors like differences in geographical location and diet, will significantly impact gut composition beyond the therapy or model of study. More causative studies must be performed for the role of the gut microbiota in neurodegenerative or neurodevelopmental disease and vice versa: how CNS diseases themselves in-turn may influence the composition of the gut microbiota. In addition, there exists an exciting possibility to leverage growing knowledge of gut bacteria to serve as predictive biomarkers of disease; however more diverse cohorts of patients must be explored to validate the translatability of microbial biomarkers across different ethnic and geographical groups.

## Targeting the microbiome-gut-brain-axis

Already, intervention strategies targeting the gut microbiota and their subsequent translation into clinical trials are underway (Table 1).

**Table 1:** Gut brain axis in clinical trials.

Trial ID	Study Title	Neurological Conditions	Interventions	Status
**NCT03808389**	Fecal Microbiota Transplantation for Parkinson's Disease	Parkinson Disease	Biological: Allogenic & Autologous FMT	Recruiting
**NCT03631823**	Gut Microbiota and Glioblastoma Multiforme Prognosis	Glioblastoma Multiforme	Drug: temozolomide with Chemotherapy	Unknown†
**NCT03575195**	Microbiota Intervention to Change the Response of Parkinson's Disease (MICRO-PD)	Parkinson Disease	Drug: Rifaximin	Recruiting
**NCT04315922**	Multiomics Targeting Microbiome Associated Changes in Stroke Patients	Stroke	Diagnostic Test: Microbiome and Plasma Characterization	Recruiting
**NCT03982290**	Psychophysiological Effects of Lactobacillus Plantarum PS128 in Preschool Children with Autism Spectrum Disorder	Autism Spectrum Disorder	Probiotic: Lactobacillus plantarum PS128 Other: microcrystalline cellulose	Unknown†
**NCT03237078**	Lactobacillus Plantarum PS128 in Patients with Major Depressive Disorder and High Level of Inflammation	Major Depressive Disorder Inflammation	Probiotic: Lactobacillus plantarum PS128	Unknown†
**NCT03279224**	Safety and Efficacy of Fecal Microbiota Transplantation in a Population with Bipolar Disorder	Bipolar Depression	Biological: Allogenic & Autologous FMT	Recruiting
**NCT03835468**	The Role of the Microbiota-gut-brain Axis in Brain Development and Mental Health	Anxiety; Emotion Regulation Abilities	Dietary Supplement: Galacto-oligosaccharides (Prebiotic)	Recruiting
**NCT02723344**	Biological Signatures, Probiotic Among Those With mTBI and PTSD	Mild Traumatic Brain Injury Post Traumatic Stress Disorder	Probiotic: L. reuteri; DSM 17938 with Sunflower & medium chain triglyceride oils	Completed August 2020
**NCT03370458**	Lactobacillus Plantarum DR7 for Gut-Brain-Axis Benefits	Stress	Probiotic: Lactobacillus plantarum DR7	Completed March 2018
**NCT03847714**	Probiotics in Dementia	Dementia; Alzheimer Disease	Dietary Supplement: Omni-Biotic Stress Repair	Recruiting
**NCT03851120**	Brain Probiotic and LC-PUFA Intervention for Optimum Early Life	Infant Cognitive Development	Probiotic and LC-PUFA with Psychosocial stimulation and healthy eating education	Recruiting
**NCT04167995**	Assessment of Probiotics Lactobacillus in the Management of ADHD	ADHD	Probiotic Formula lacteal forte	Unknown†
**NCT02817074**	MIND Diet Intervention and Cognitive Decline	Cognitive Decline	Diet: Mediterranean-DASH	Active, Not Recruiting
**NCT03679533**	The Impact of Cranberries On the Microbiome and the Brain in Healthy Ageing study (COMBAT)	Aging Cognitive Decline	Dietary Supplement: Freeze-Dried Cranberry Powder	Completed September 2020

† Study has passed its completion date and status has not been verified in more than two years.

**Fecal Microbiota Transplantation (FMT)** was shown to prevent disease progression of MS^56^ and seizures^57^ for a significant duration as well as temporarily ameliorate PD symptoms (e.g. leg tremors and neuroinflammation).^58^ Moreover, feces transplanted from senescence-resistant mice to AD mice models showed improvement of spatial learning and memory.^59^ However, FMT is still clouded with uncertainties from imprecise definitions of a favorable microbiota and a need to validate its benefits and long-term effects, especially considering its ability to transfer both immune-regulatory and disease-promoting bacteria. To address this, several clinical trials have been launched, including one titled “Safety and Efficacy of Fecal Microbiota Transplantation in a Population with Bipolar Disorder,” which sought to simultaneously investigate the effects of FMT on clinical parameters associated with depression and anxiety as well as its safety and tolerance for use in patients. Overall, the large potential of FMT in providing a positive change to the gut microbiota composition of diseased patients is offset by its lack of specificity. Moving forward, it remains to be seen whether FMT will suffice as a stand-alone therapy; likely, the answer to that will depend upon the specific nature of the disease and the bacteria that modulate them.

**Antibiotics** play an important role in changing the composition of gut bacteria and thus, influencing immune response and therapeutic efficacy. In gliomas, for example, antibiotics are essential for preventing or treating life-threatening infections in patients resulting from microbial-induced immunosuppression and other detrimental effects caused by harmful bacteria; more broadly, targeting the gut microbiome using appropriate antibiotics may allow for improved tumor control. Antibiotics may even be used as a direct therapy, with the antibiotic clofoctol recently being demonstrated to reduce glioma growth via inhibition of glioma-stem-cell proliferation through upregulating Krüppel-like factor 13, a tumor suppressor gene.^60^ However, significant caution must be taken as antibiotics can cause collateral damage to the diversity of the gut microbiota and, consequently, neurological diseases. Separate studies demonstrated that broad-spectrum antibiotic treatment decreased cognitive function^61^ in wild-type mice as well as increased mortality rates and occurrences of severe colitis in mice following stroke inductions.^59^ Another study noted increased prevalence of vancomycin-resistant bacteria following antibiotic administration, such as *Enterococcus faecium,* which increased risk of bloodstream infections and patient mortality.^62^ Moreover, a study examining treatment of antibiotics on syngeneic glioma mouse models concluded that antibiotic treatment contributed to increased intracranial glioma growth and significantly altered microbiota composition, weakening the brain's immune capacity to fight tumors. Specifically, the study observed early impairment of cytotoxic NK cells, altered inflammatory and homeostatic protein expressions, and reduced species diversity of *Prevotellaceae, Rikenellacaea*, and *Helicobacteraceae* families.^63^ Clinically, “Microbiota Intervention to Change the Response of Parkinson's Disease” was recently launched to investigate the impact of Rifaximin, an antibiotic medication, in reducing potentially deleterious bacterial populations and alleviating PD clinical symptoms, including inflammation serum markers. Overall, it is apparent that there is a need to harness knowledge and impacts of the microbiota with any new antibiotic or medication.

**Figure 3:**
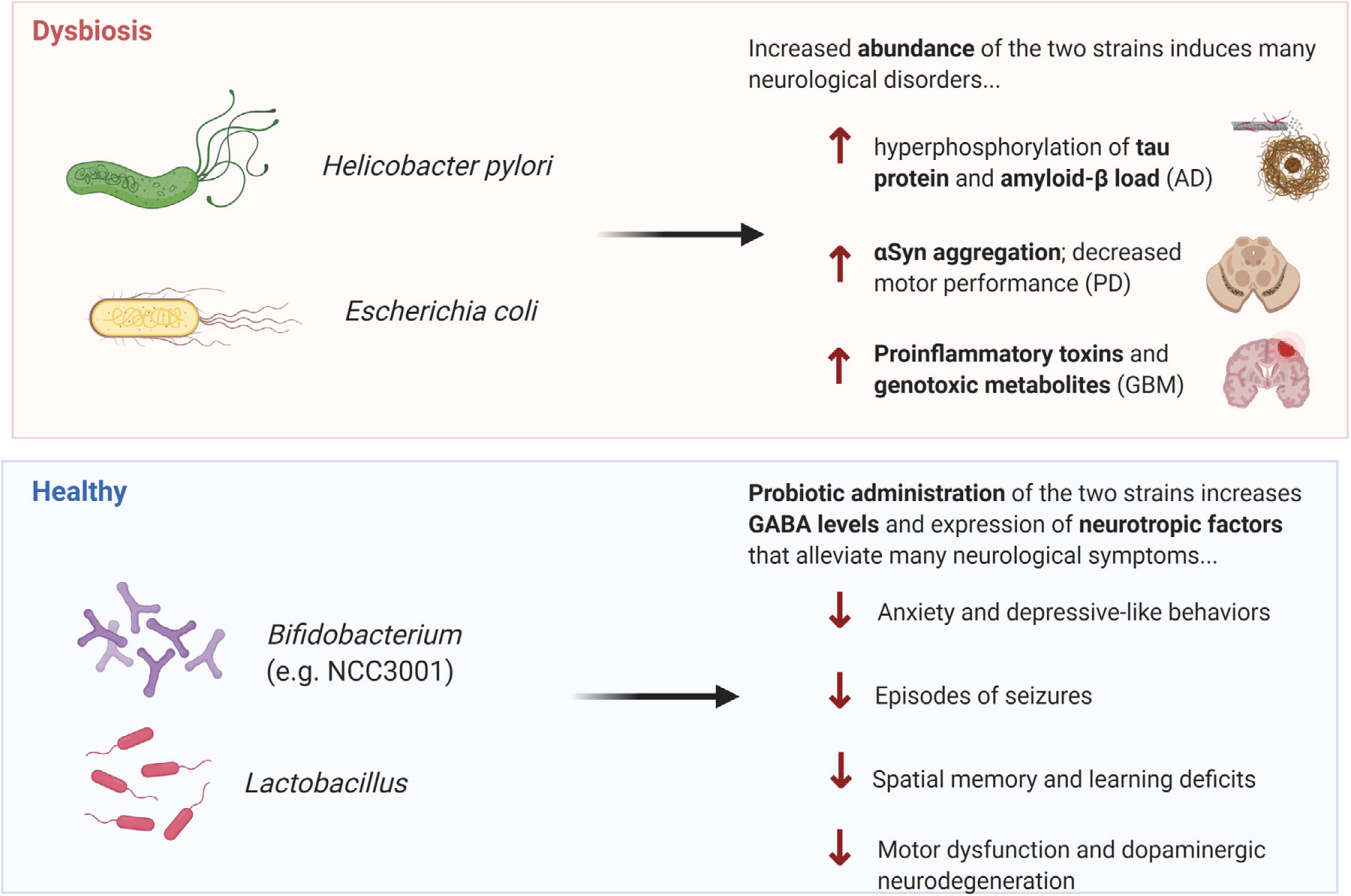
Microbials modulate the development and treatment of CNS disorders. Microorganisms can promote production of essential metabolites, neurotransmitters, and other neuroactive compounds that influence the progression or treatment of various CNS diseases. In the setting of dysbiosis, increased prevalence of *Helicobacter pylori* and *Escherichia coli*, for example, was shown to induce the progression of many neurological disorders and symptoms including the hyperphosphorylation of tau protein and amyloid-Beta load (indicative of Alzheimer's disease), α-Synuclein aggregation and decrease motor performance (indicative of Parkinson's disease), and increased proinflammatory toxins and genotoxic metabolites (indicative of CNS malignancies). Conversely, probiotic administration of beneficial strains such as *Bifidobacterium* and *Lactobacillus*, for example, have been shown to alleviate many neurological symptoms through increase GABA levels and expression of neurotrophic factors. Effects include decreased anxiety and depressive-like behaviors, decrease episodes of seizures, decreased spatial memory & learning deficits, and decrease motor dysfunction & dopaminergic neurodegeneration.

**Probiotics** are often components of food products or supplements that we consume daily, such as yogurt. Increasingly, more “designer probiotics” have been introduced that are genetically engineered to maximize the beneficial effects of select bacteria and are often included in more traditional pharmaceutical routes.^64^ Importantly, multiple studies have demonstrated beneficial effects in probiotic administration of *Bifidobacteria* and *Lactobacillus* – which promotes GABA levels and expression of neurotropic factors – including reduced occurrences of seizure episodes in patients with drug-resistant epilepsy*,*^65^ amelioration of deficits in spatial memory and learning for patients with AD (via mixture with fermented milk),^66^ and reduction of motor dysfunction and dopaminergic neurodegeneration in MitoPark PD mouse models^67^ (Figure 3). Furthermore, administration of SLAB51, a new probiotic formulation, in a human *in vitro* PD model (SH-SY5Y) was able to increase cell viability via reducing oxidative stress and neuronal death as well as reverse phenotypic deficits in motor behavior when placed in a rodent PD-model.^68^ Of the numerous probiotics being studied, most studies are focusing on the effects of probiotic *Lactobacillus Plantarum* in several CNS diseases, including major depressive disorder (Identifier: NCT03237078), ADHD (Identifier: NCT04167995), ASD (Identifier: NCT03982290), and stress (Identifier: NCT03370458). Overall, the results to date convey great potential in harnessing beneficial gut bacteria as novel therapeutic options for neurological diseases. However, there is wide variability in composition, stability, and authenticity of probiotics with no consensus on dosage, duration, and specific strains to use; moreover, host colonization resistance brings a notable concern with any use of probiotic-based therapies. Thus, caution must be exercised in any use of probiotics to patients until more holistic characterization of these bacteria are conducted. Admittedly, while beneficial effects are not durable as probiotics generally cannot colonize in the acidic environment of the gut for a long time,^4^ it may arguably be a therapeutic advantage for probiotics to not permanently alter the cell environment and simply only require more frequent consumption as needed.

**Diet**, though not necessarily a therapeutic treatment, also plays a significant role, especially as accumulating evidence indicate that intensive changes to dietary regimens can rapidly alter gut microbial profiles. In fact, a specific dietary class has been recognized as **prebiotics,** which are indigestible fibers (e.g., fructo-oligosaccharides, galacto-oligosaccharides, and resistant starch) selectively utilized by gut microorganisms as the primary nutrition source for conferral of health benefits. Commonly found in fruits, vegetables, grains, and human milk, prebiotics have the advantage of modulating the gut microbiota more globally. Reductions in dietary fiber, for example, significantly decreases the abundance of immune-promoting *Faecalibacterium prausnitzii* and SCFA butyrate, which help support digestive health. Conversely, a high-fat, low-fiber animal-based diet decreased levels of beneficial metabolic bacteria and increased bile-tolerant microorganisms*.*^69^ Related to this, there are **synbiotics**, which are a combination of probiotics and prebiotics in which prebiotics are used to supplement (providing a source of fermentable fiber) and enhance the viability of the probiotic,^4^ and **postbiotics**, which entail metabolites of bacterial fermentation including functional bioactive compounds such as SCFAs and gut peptides. While there is still a need for more elucidated targets, the fact that postbiotics are nonviable entities provides a distinct advantage over probiotics in terms of increased shelf life and safety profile.^70^ Importantly, it has been observed that higher-fiber diets resulted in greater diversity richness of the gut microbiota and a higher abundance of favorable taxa as well as increased butyrate production, which plays neuroprotective roles and enhances neuron plasticity. Not surprisingly, diet plays a significant role in outcomes associated with CNS disorders. In a study conducted in PD mice, for example, the experimental group that received a fasting-mimicking diet had significantly less motor function decline and dopaminergic neurons loss compared to control.^71^ Moreover, in epilepsy patients, ketogenic diets composed of high-fat, low-carbohydrate, and adequate-protein were found to reduce the number of seizures through increasing GABA and glutamate levels.^72^ Interestingly, these findings are translating across numerous diseases, including gliomas, with one pre-clinical study noting massive tumor cell death upon the simultaneous treatment of mice using a calorically restricted ketogenic diet and 6-diazo-5-oxo-L-norleucine, a glutamine antagonist, with no obvious toxicity.^73^ To translate from bench to bedside, numerous clinical studies are currently being conducted to further investigate the role of diet in varying neurological disorders. A notable ongoing longitudinal study titled “The Role of the Microbiota-gut-brain Axis in Brain Development and Mental Health”, for example, is investigating how prebiotic intake (Galacto-oligosaccharides) affects cortical excitability & plasticity as well as regulation of anxiety & thought control abilities. Another study, titled “Mediterranean-DASH Diet Intervention for Neurodegenerative Delay”, is now in a Phase III clinical trial investigating the effects of a hybrid diet on the cognitive decline of elderly, overweight individuals with poor prior nutritional intake. Despite the lack of precision in targeting specific gut microbes and need for a better understanding of how starting states of the commensal bacteria populations may influence efficacy of dietary mediations, there is a clearly increased interest of benefits that healthy diets convey for CNS therapy interventions and outcomes.

The ability to unravel the interactions between the microbiome and therapeutic response will likely catalyze the next big change in the discovery pipelines of therapeutic developments; it is not a stretch to say that it may soon be routine for human-directed drugs to be screened for impacts on microbial populations.

## Perspectives

The evolution of the microbiome field has enabled a significant step closer to understanding CNS disease treatments through untangling the intricate networks of the immune system, gut microbiota, and microbiota-gut-brain axis. Moving forward, it will be important to recognize that the microbiome may be a key contributor to the prevention, development, treatment, and management of CNS diseases, where something as simple as an every-day diet may pose significant therapeutic potential especially when synergized with existing therapies. Moreover, with increased knowledge on the gut microbiota, new strategies will become available for non-invasive predictors of prognosis and predictive biomarkers (e.g., quantifying community richness and proportions of “beneficial” to “harmful” bacteria via fecal DNA sequencing) for a variety of CNS disorders and respective outcomes. Ultimately, the pliability of the microbiota promises exciting potential and hope for ushering in a new frontier for precision medicine.

## Outstanding questions

There remain key reservations and unknowns about the MGBA founded upon the interwoven complexities that make up human neurological diseases. For one, gut microbial profile varies widely between individuals and even if two individuals harbor the same bacterial species, they could vary in strain that possess different functionalities within the host. Additionally, within the individual, there is a duplicity in microbial roles to physiological health such that the same microbe could both harm and benefit human health. Secondly, as existing animal models are limited in their ability to replicate the totality of any human disease, the reproducibility of results observed preclinically in mostly rodent models remains largely unanswered, posing obstacles to the successful translation to the bench side. Moreover, with the variety of potential mechanisms for targeting the gut-brain-axis, there remains a crucial need to separate correlation from causality and caution must be employed to ensure no off-side effects. Of the limited existing preclinical data, very few microorganisms (e.g., *Lactobacillus* and *Bifidobacterium* spp.) display reproducible and robust benign physiological effects and even fewer still are actively involved in clinical trials (of which results largely remain inconclusive). With the increasing presence of many commercially available over-the-counter antibiotics and dietary regimens boasting of probiotics, a call must be made for greater public awareness in how they may ultimately impact our gut microbiome and, consequently, our health.

## Declaration of interests

K.S. owns equity in and is a member of the Board of Directors of AMASA Therapeutics, a company developing stem cell–based therapies for cancer. K.S.’s interests were reviewed and are managed by Brigham and Women's Hospital and Partners HealthCare in accordance with conflict-of-interest policies.
